# Prevalence of Rift Valley Fever among Ruminants, Mayotte

**DOI:** 10.3201/eid1806.111165

**Published:** 2012-06

**Authors:** Catherine Cêtre-Sossah, Aurélie Pédarrieu, Hélène Guis, Cédric Defernez, Michèle Bouloy, Jacques Favre, Sébastien Girard, Eric Cardinale, Emmanuel Albina

**Affiliations:** Centre de Coopération Internationale en Recherche Agronomique pour le Développement, Montpellier, France (C. Cêtre-Sossah, A. Pédarrieu, H. Guis, E. Albina);; La Direction de l’Agriculture et de la Forêt de Mayotte, Mamoudzou, Mayotte (C. Defernez, J. Favre);; Institut Pasteur, Paris, France (M. Bouloy);; Centre de Coopération Internationale en Recherche Agronomique pour le Développement, Sainte-Clotilde, France (S. Girard, E. Cardinale)

**Keywords:** Rift Valley fever, arbovirus, Mayotte, viruses, Rift Valley fever virus, ruminants, RVFV

## Abstract

Rift Valley fever threatens human and animal health. After a human case was confirmed in Comoros in 2007, 4 serosurveys among ruminants in Mayotte suggested that Rift Valley fever virus had been circulating at low levels since 2004, although no clinical cases occurred in animals. Entomologic and ecologic studies will help determine outbreak potential.

Rift Valley fever virus (RVFV) usually causes large, explosive epidemics among animals and humans and circulates in many African countries and the Arabian Peninsula (*1**–**3*). The human and veterinary medical role of this mosquito-borne virus was highlighted at the end of 2006 and early 2007, when a large epidemic/epizootic occurred in eastern Africa (*4**,**5*) and Madagascar, during 2 successive rainy seasons (*6**,**7*). More recently, South Africa and Mauritania were severely affected (*8**,**9*). This wide dissemination potential emphasizes that Rift Valley fever constitutes a threat for human and animal health on the African continent and beyond. In Mayotte in July 2007, recent RVFV infection was detected in a 12-year-old boy with a severe neuroinvasive illness. This patient had recently arrived from Grande Comore, Union of the Comoros, where RVFV circulation had been confirmed (*10**–**12*).

Starting in April 2008, given the proximity of Comoros and Mayotte and considering the risk for introducing RVFV by illegal animal movements, active laboratory-based surveillance for Rift Valley fever was implemented among susceptible ruminants in Mayotte. A series of 4 serosurveys was designed to clarify the epidemiologic situation. The first survey captured information about goats and cattle illegally introduced to the northern part of the island of Mayotte, the site of most illegal imports because of its proximity with the Comoros island of Anjouan (Figure 1). The second survey was a retrospective islandwide serologic survey of ruminant samples collected during 2007–2008, intended to capture a broader view of the situation. The third survey was a 4-year retrospective serosurvey of ruminant samples collected during 2004–2007, intended to increase knowledge of the history of the virus on the island. The fourth survey, a longitudinal serologic study on goat farms, assessed whether the virus was still circulating in 2008.

**Figure 1:**
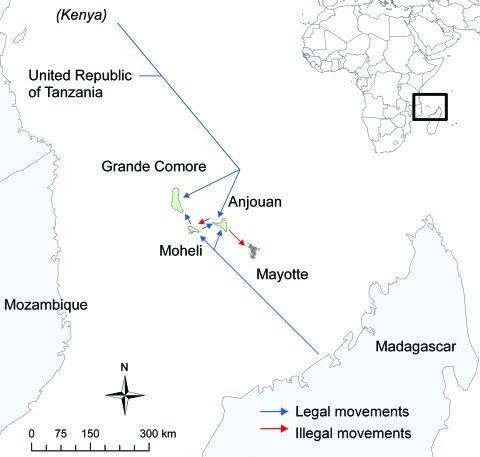
Potential legal and illegal movements of animals around the Comoros and Mayotte.

## The Study

The first survey, intended to clarify the Rift Valley fever epidemiologic situation on the island, was undertaken in the M’Tsangamouji area (northern part of Mayotte). It examined samples from 29 illegally introduced goats and 79 cattle born on the island and living near the goats. Among the 29 goats, competitive IgG ELISA found IgG against RVFV in 4 goats that had been introduced illegally during November 2007–April 2008 (*13*), and IgM-capture ELISA found IgM against RVFV in 2 goats (*14*), suggesting recent infection. Among the 79 cattle, IgG against RVFV was found in 29 (37%) and IgM against RVFV was found in 3 (4%).

**Figure 2:**
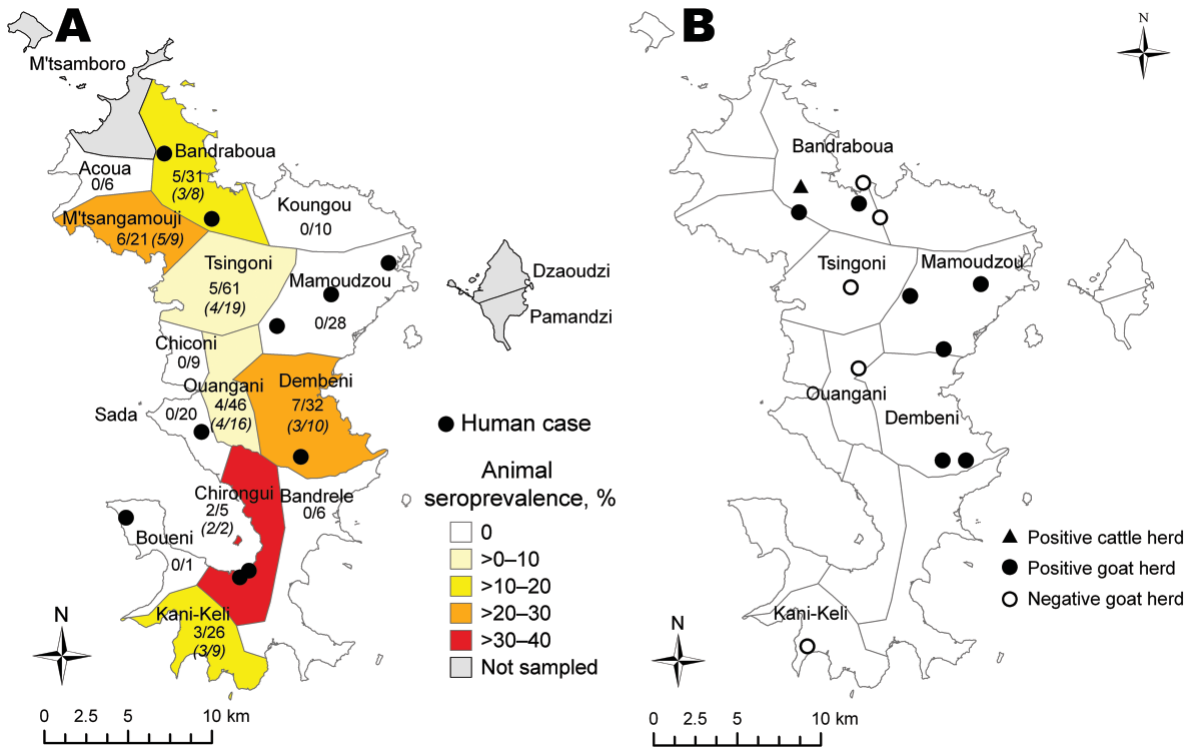
Rift Valley fever in Mayotte, by municipality. A) Human cases and animal and herd seroprevalence. Values under municipality names are seroprevalence by herd (no. infected herds/no. herds) and, in parentheses, by animal in infected municipalities (no. infected/total no.). B) Status of goat herds sampled for longitudinal serologic study, Mayotte, 2008–2009.

These data led us to conduct the second survey, a retrospective study on the whole island to define the geographic distribution of the infection and to trace back the period of introduction. This survey analyzed 301 cattle serum samples collected during June 2007–May 2008 on 104 farms in 17 districts. Exposure to RVFV was indicated by competitive IgG ELISA detection of RVFV-specific antibodies. Positive results were found for 32 samples from cattle in 9 districts (Table). The overall apparent RVFV seroprevalence of 10.6% (95% CI 7%–14%) was supported by the high specificity of the ELISA (*14*). The 32 positive samples came from cattle distributed all over the island (Figure 2, panel A).

**Table:** Rift Valley fever virus seroprevalence among cattle, Mayotte, June 2007–May 2008

District	No. positive/no. tested	Seroprevalence, %
Acoua	0/6	0
Bandraboua	5/31	16.13
Bandrele	0/6	0
Boueni	0/1	0
Chiconi	0/9	0
Chirongui	1/3	33.33
Dembeni	7/32	21.88
Dzoumogne	0/1	0
Kahani	1/5	20
Kani Keli	3/26	11.54
Koungou	0/10	0
Mamoudzou	0/28	0
Mirereni	1/2	50
Mtsangamouji	6/21	28.57
Ouangani	3/40	7.50
Sada	0/20	0
Tsingoni	5/60	8.33
Total	32/301	10.63

Because RVFV circulation had been confirmed as early as 2007–2008 in Mayotte, a third cross-sectional and retrospective study was conducted to trace previous virus circulation. The 120–130 samples that had been collected from cattle since 2004 were randomly selected every year over a 4-year period and analyzed by IgG ELISA; results were confirmed by neutralization tests (*15*). These results helped evaluate RVFV circulation on Mayotte island before the 2007–2008 outbreak on the eastern Africa mainland. In 2004, a total of 29 of 130 cattle had IgG against RVFV; thus, seroprevalence was high (22.66%). In 2005, seroprevalence rates fell; IgG against RVFV was found in only 4 of the 130, suggesting low levels of RVFV circulation. In 2006 and 2007, seroprevalence increased; IgG against RVFV was found in 16 of 130 and 39 of 126 cattle, reaching seroprevalence rates of 12.31% and 30.95% for 2006 and 2007, respectively. Specific IgM against RVFV was not detectable during this cross-sectional and retrospective study.

The fourth survey, intended to evaluate the recent virus dynamics in Mayotte, was a longitudinal serologic survey of goat farms. In June 2008, a total of 13 goat farms were selected and all 272 animals were screened for antibodies against RVFV. Of the 13 farms, 8 had seropositive animals (herd prevalence 62%, 95% CI 35%–88%) (Figure 2, panel B). Intraherd prevalence ranged from 6% to 42%. The 5 farms with seronegative goats (total 70 goats) were included in the longitudinal study. During August 2008–August 2009, the seronegative goats were sampled every 6–8 weeks. Only 1 goat, located in Bouyouni and sampled in February 2009, had seroconverted and was confirmed IgM positive for RVFV. Virus isolation attempts were unsuccessful.

## Conclusions

The 4 serologic surveys conducted in Mayotte revealed medium to high rates of RVFV prevalence all over the island. The high rates obtained with the first survey in the M’Tsangamouji area suggest that illegal animal movements from the Comoros are a likely source for RVFV introduction onto Mayotte. Results of the 4-year survey show that the virus was already present in 2004. After a low seroprevalence rate in 2005, the increased seroprevalence rates for 2006 and 2007 suggest that the virus had recirculated or had been newly introduced.

It is unclear why relatively high circulation of RVFV in Mayotte and an increased rate of seroprevalence to 22% did not result in detectable clinical cases in animals while Rift Valley fever was diagnosed for humans with brain disorders (*11*). This finding might be because the density of susceptible animals on the island was high enough to support virus circulation but too low to support waves of epidemic abortion and death. These study findings, coupled with epidemics in eastern Africa, illustrate the risk for introduction of infectious agents from the African mainland to Mayotte or other Comoros islands. Entomologic studies need to be conducted to identify all potential vector species on the island and to better understand the ecologic and climatic factors that favor RVFV dissemination. The ecologic factors in favor of Rift Valley fever outbreaks might be comparable between Mayotte, the other Comoros islands, Madagascar, and the eastern African mainland (Kenya, Tanzania, and Mozambique) but need to be looked at more closely. In Mayotte, an entomologic surveillance program is being developed to help define the distribution of potential vectors in association with virus circulation and provide better understanding of disease spread mechanisms. The role of wildlife should also be investigated. These data highlight the need for extensive studies to determine RVFV distribution and to evaluate the effect of Rift Valley fever on the susceptible livestock populations.
